# ﻿Gut microbiome composition of Trichoptera larvae across functional feeding groups: a case study from the Provo River, Utah, USA

**DOI:** 10.3897/zookeys.1263.147980

**Published:** 2025-12-10

**Authors:** Gabriela Jijón, Connor Hough, Danyon Gedris, Paul B. Frandsen, John M. Chaston

**Affiliations:** 1 Department of Plant and Wildlife Sciences, Brigham Young University, Provo, UT 84602, USA Brigham Young University Provo United States of America; 2 Department of Biology, Brigham Young University, Provo, UT 84602, USA Brigham Young University Provo United States of America; 3 Bean Life Science Museum, Brigham Young University, Provo, UT 84602, USA Brigham Young University Provo United States of America

**Keywords:** 16S metabarcoding, aquatic insect larvae, gut microbial community characterization, host feeding ecology, preliminary study, temporal differences

## Abstract

Diet is known to be a major factor in the gut microbiome of many groups of insects. Larvae from the insect order Trichoptera have varied feeding behaviors, encompassing all functional feeding groups, making them an excellent group for studying the links between diet and gut microbe community variation. However, no previous study has examined these links in caddisflies. Here, we characterize the gut microbiome composition of four caddisfly genera belonging to four different functional feeding groups over two sampling periods using 16S metabarcoding. We found that the sampling date had the strongest influence on gut microbiome variation. Host functional feeding groups and phylogeny also influenced gut community composition; however, improved sampling is necessary to confirm this relationship. Our preliminary results point to interesting differences among larvae from different feeding groups and suggest future areas for research, including performing species-level identification of the caddisfly larvae, using more taxa within and between functional feeding groups, using temporal and larval stage-matched replicates, assessing the degree of microbiome residency in caddisfly guts, and performing deeper sequencing.

## ﻿Introduction

Some insects depend on symbiotic relationships with bacteria to survive and function (Gupta and Nair 2020), while others exhibit a lower reliance on microbes (Hammer et al. 2017, 2019). Microbes located in the gut play an important role in physiology and digestion for some insects (Moran et al. 2013; Krishnan et al. 2014; Munoz-Benavent et al. 2021). For others, microbes may be transient in the gut and have a minimal functional role (Hammer et al. 2017, 2019). Studies on insect-associated microbiomes have primarily focused on terrestrial insects, and relatively little is known about aquatic insect gut microbiota (Men et al. 2022; Millar et al. 2022). While some previous studies have documented insect host associations, developmental stage influence, and environmental impacts on microbiome composition in aquatic insects (Castillo et al. 2020; Nobles and Jackson 2020; Millar et al. 2022), only a few studies have focused on the connection between aquatic insect diet and gut microbiota.

Since diet influences microbiome composition, evaluating microbiome differences among feeding modes could reveal possible relationships between microbes and food intake in aquatic larvae. To understand the complex links between organisms in aquatic food webs, freshwater biologists characterize aquatic insects into functional feeding groups (FFGs), which are defined by morphological and behavioral adaptations of aquatic macroinvertebrates to different food sources available in freshwater environments (Merritt et al. 2017). The feeding categories include filtering collectors, gathering collectors, shredders, scrapers, piercers-herbivores, and predators (Cummins and Klug 1979; Merritt et al. 2017). Larvae from the highly diverse insect order Trichoptera, also known as caddisflies, are unusually ecologically diverse and encompass all functional feeding groups, consuming a large variety of in-stream food sources (Cummins and Klug 1979; Wiggins and Currie 2008; Morse et al. 2019).

Despite their diversity in both species number and feeding types, there are no studies examining the microbiomes of larval caddisflies across varying feeding modes. Given the diversity of feeding types across Trichoptera, caddisfly larvae can serve as models for investigating the relationships between diet and gut microbiota in aquatic insects. Here, we aim to provide a preliminary overview of the gut microbiome composition of caddisflies that possess diverse feeding behaviors by characterizing the gut microbiomes of 61 caddisfly larvae belonging to four genera and representing four functional feeding groups.

## ﻿Methods

### ﻿Sample collection

We collected 61 caddisfly larval samples, 31 samples in the fall of 2022 (30 September) and 30 in the winter of 2023 (4 February) in the Provo River, Provo, Utah (40°15.91'N, 111°39.81'W), using D-net samplers. We field sorted the benthic samples and placed the live caddisfly specimens in containers with stream water. We transported the live organisms into the laboratory and placed them in dechlorinated tap water without food. We identified the larvae taxonomically at the genus level and classified them into a primary functional feeding group using Merritt and Cummins (1996). We performed gut dissections the following day. Larval specimens of *Oecetis* sp. and *Tinodes* sp. were too small to dissect, so we sampled the whole body (Suppl. material 2: table S1). From the fall collection, we sampled 12 *Hydropsyche* sp. (filtering collector), 10 *Oecetis* sp. (predator), and 9 *Hesperophylax* sp. (shredder) larvae. From the winter collection, we sampled 11 *Hydropsyche* sp. (filtering collector), 13 *Tinodes* sp. (scraper), 4 *Hesperophylax* sp. (shredder), and 2 *Oecetis* sp. (predator) larvae. All genera sampled represented a unique functional feeding group, and *Tinodes* sp. larvae were collected only in the winter.

### ﻿DNA extraction and sequencing

The V4 region of 16S rRNA was sequenced according to Kozich et al. (2013) for microbiota characterization. We extracted and sequenced the DNA for samples from each collection date separately. For DNA extraction, we used the Zymo Quick-DNA Fecal/Soil Microbe 96 Kit (Zymo Research, D6011). We placed the dissected gut or whole larval samples in tubes with 400 µL of BashingBead buffer and stored them at −20 °C until DNA extraction. The guts (and bodies when dissections were not possible) were homogenized using a 2010 Geno/Grinder (SPEX SamplePrep, Metuchen, NJ, USA) for 2 min at 1750 RPM. We followed the manufacturer’s instructions, including adding 0.5% (v/v) beta-mercaptoethanol to the genomic lysis buffer, and DNA was eluted in 50 µL Elution Buffer. We stored the extracted DNA at −20 °C until PCR amplification.

Libraries for 16S rRNA V4 sequencing were prepared as 20 µL PCR reactions using AccuPrime™ Pfx DNA Polymerase (ThermoFisher, 12344032) and dual-barcoded primers as in Kozich et al. (2013). We added 2 µL template DNA, 1 µL each of 10 µM primer (Suppl. material 2: table S1), and 0.25 µL polymerase per reaction (all else as in manufacturer's instructions). We included a reagent-only control for the DNA extractions, nuclease-free water and positive controls for the PCR reactions for the February samples. We then performed a 1% agarose gel electrophoresis on a random subset of samples to assess amplification success for the February samples. Amplification was visible for the subset of the samples (Suppl. material 1), and the negative PCR controls did not produce any visible bands in the gel electrophoresis.

For PCR product normalization, we followed the manufacturer’s instructions when using the Just-a-Plate 96 PCR Normalization and Purification Kit (Charm Biotech, JN12010). All samples for each run were pooled together in equal volumes and concentrated to 12 µL using the gDNA Clean & Concentrator -5 Kit (Zymo Research, 11-302C). We selected DNA fragments by size using a Select-a-Size DNA Clean & Concentrator Kit (Zymo Research, D4080), following the manufacturer’s double-size selection protocol. Fragments of ≤ 200 bp and ≥ 700 bp were depleted. Fragment sizes were evaluated using an Agilent Bioanalyzer (Agilent Technologies, Santa Clara, CA, USA), followed by a 250–450 bp fragment size selection using a Sage Science Blue Pippin (Sage Science, Beverly, MA, USA), and the molarity of the pooled samples was assessed with qPCR at BYU DNA Sequencing Center in Provo, Utah, USA.

Each pool was sequenced on a partial Illumina MiSeq run, pooled with other samples to target ~10,000 reads per sample. Before sequencing, we diluted each final library to 4.0 pM and pooled it with 15% PhiX. We loaded 600 uL to the MiSeq Reagent Kit v2 500 cycle cartridge (Illumina, MS-102-2003). Custom read1, read2, and index sequencing primers were loaded to the cartridge as described by Kozich et al. (2013).

### ﻿Sequence analysis

We used QIIME 2 v. 2023.2 (Bolyen et al. 2019) to analyze the 16S rRNA V4 region sequencing data on the raw sequencing reads that passed Illumina quality filters from each sequencing run. See Suppl. material 2: table S1 for the metadata table used for downstream processing.

Sequence analysis, including denoising, sequence identification and amplicon sequence variant (ASV) assignments, were performed using QIIME2 (Bolyen et al. 2019). Denoising was performed using Divisive Amplicon Denoising Algorithm (DADA2) v. 1.26.0 (Callahan et al. 2016). qiime dada2 denoise-paired (parameters = --p-trunc-len-f, --p-trunc-len-r) to filter phiX reads and trim Illumina amplicon sequences on the .qza files. Based on quality scores (≥30), the sequences were trimmed at 240 and 180 base-pair lengths for forward and reverse sequences, respectively, for both sampling dates. The forward and reverse-complement reads were merged using the default settings (at least identical 12 base-overlap) (Callahan et al. 2016). We used the GreenGenes classifier database 13_8_99 (McDonald et al. 2012) to assign microbial taxa to ASVs, and then chimeras were identified and removed (Callahan et al. 2016). We excluded any feature (i.e., ASV) that was classified as Archaea, Chloroplast or Mitochondria (parameter = --p-exclude Archaea,Chloroplast,Mitochondria).

Alpha and beta diversity metrics were generated for the combined dataset (including both dates) as well as separately for the September and February datasets. These metrics were generated with qiime diversity core-metrics-phylogenetic using a sampling depth of 412 (parameter = --p-sampling-depth) for all datasets. We carried out a non-metric dimensional scaling dissimilarity (NMDS) analysis using metaMDS() (vegan v. 2.6-10; Oksanen et al. 2025) and PERMANOVA tests using qiime diversity beta-group-significance (parameter = --p-pairwise) on 999 permutations by collection date and FFG/taxonomic classification based on Bray-Curtis dissimilarity distances for the combined and separate September and February datasets. We ran qiime diversity alpha-rarefaction using 2,000 for the --p-max-depth option and resamplings of 10 (--p-iterations) to produce the alpha rarefaction plot for the combined dates and qiime taxa barplot to generate taxon bar plots of the combined and separate sampling dates.

## ﻿Results

The total demultiplexed sequence counts for the forward and reverse sequences were 468,085 (Suppl. material 2: table S2). The mean count per sample for the forward and reverse sequences was 7,673.52. Post filtering, 420,682 sequences remained, with an average of 6,896.43 sequences per sample (Suppl. material 2: table S3). A total of 404,087 non-chimeric, denoised, and merged sequences were obtained, with an average of 6,624.38 sequences per sample (Suppl. material 2: table S3). After filtering for non-bacterial sequences, 1,360 unique ASVs and 381,990 sequences were recovered. At the set sampling depth (412), five samples from February 2023 and one from September 2022 were discarded, retaining 5.93% (22,660) ASVs and 90.16% (55) of the samples for the combined dataset. The September 2022 dataset retained 3.46% (12,360) ASVs and 96.77% (30) of the samples. The February 2023 dataset retained 42.08% (10,300) ASVs and 83.33% (25) of the samples.

**Figure 1.**
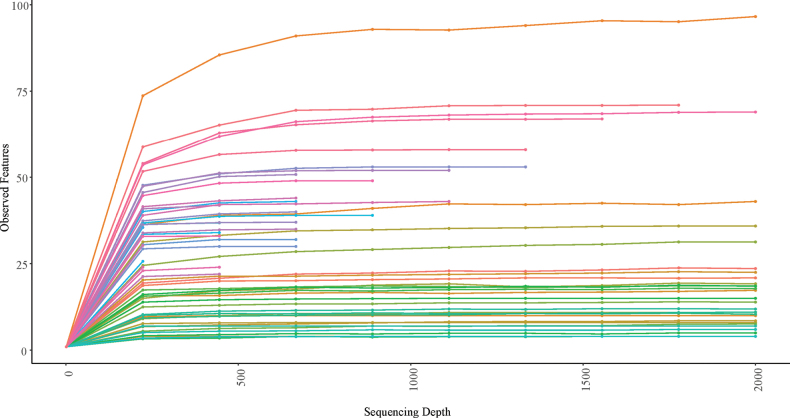
Alpha rarefaction curves for the fall and winter combined dataset. Observed features (ASVs) were plotted against sequencing depth. Maximum depth was set at 2,000 and resampling at 10.

The rarefaction curves of alpha diversity for all remaining samples reached an asymptote before the set maximum depth of 2,000 (Fig. 1). The sample showing the greatest alpha diversity was a September *Hydropsyche* sp. (ice 1412) sample. While the flatlining of the curve likely suggests we detected the most abundant organisms in the caddisflies, a deeper sequencing depth would likely have increased sensitivity and detected rarer taxa (Zaheer et al. 2018; Pereira-Marques et al. 2019).

Proteobacteria was the most abundant phylum (52.96%) across all samples when combining both collection dates, followed by Actinobacteria and Acidobacteria, with a relative abundance of 11.85 and 6.94%, respectively. The most abundant classes were Gammaproteobacteria (31.96%) and Alphaproteobacteria (12.44%), and the most abundant orders were Enterobacteriales (28.94%), followed by Actinomycetales (6.73%).

### ﻿Microbiome differences across host functional feeding groups and phylogeny

**Figure 2.**
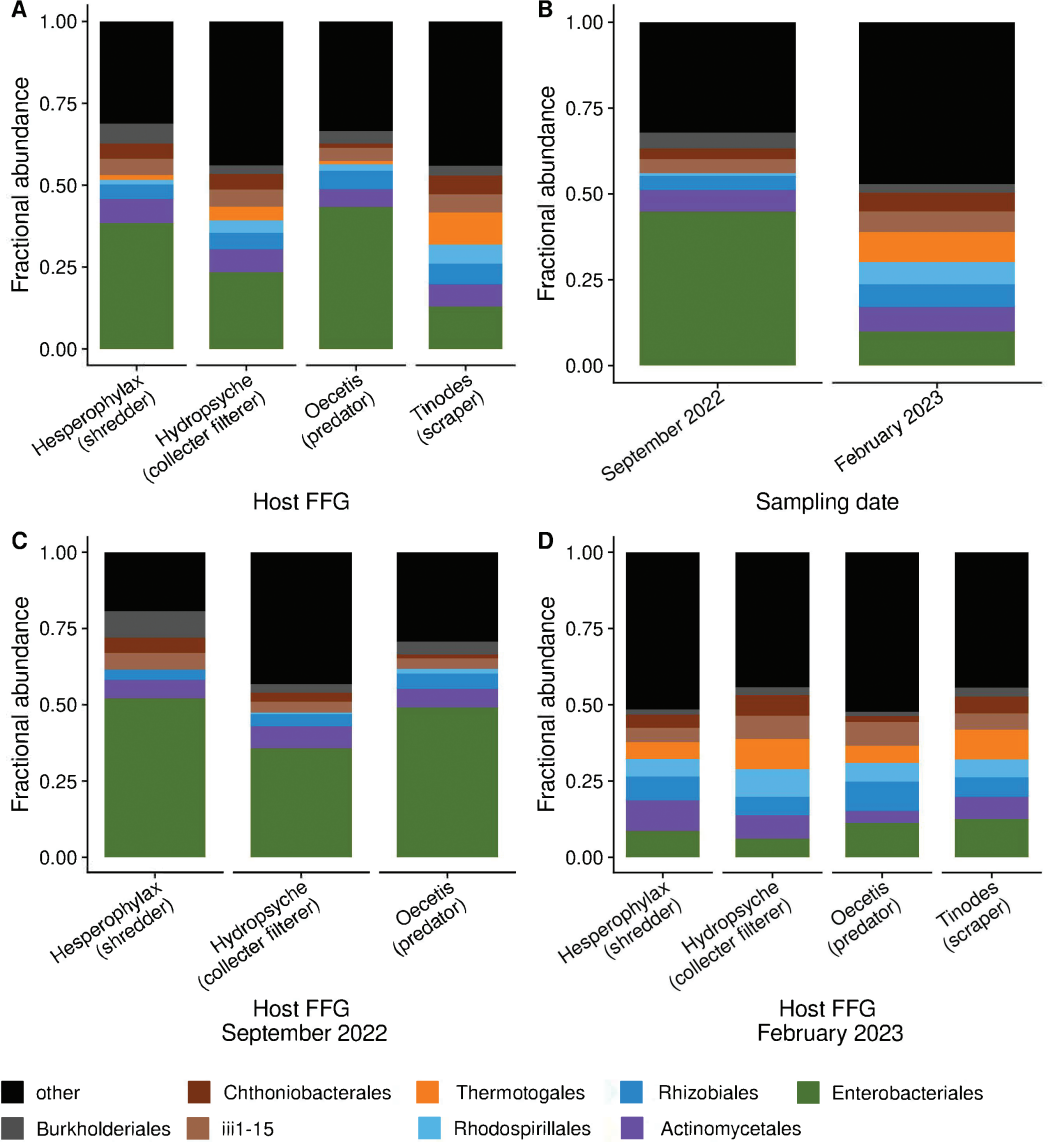
Microbiota fractional abundance taxon plots across: **A.** Functional feeding groups (FFGs) of host and **B.** Sampling date and functional feeding groups of host on the two sampling dates: **C.** September 2022 and **D.** February 2023. The legend shows abundant microbial taxa with less abundant microbiota grouped as “other” (black).

Microbiome composition showed some divergence based on host FFG and phylogeny. Enterobacteriales were the most abundant microbe for all taxa and FFGs when combining both sampling dates (Fig. 2A). Reads assigned to the Enterobacteriales comprised > 28.9% of the gut microbiota, and Actinomycetales, the second most abundant microbe, was 6.7% of the microbiota (Fig. 2A). These were the two most abundant taxa in every group except the *Tinodes* sp., where the second most abundant taxon was Thermotogales (9.7% relative abundance). It is worth noting that the gut microbiome of a September *Hesperophylax* sp. sample (ice1439) was composed almost entirely (>99%) of Proteobacteria.

**Figure 3.**
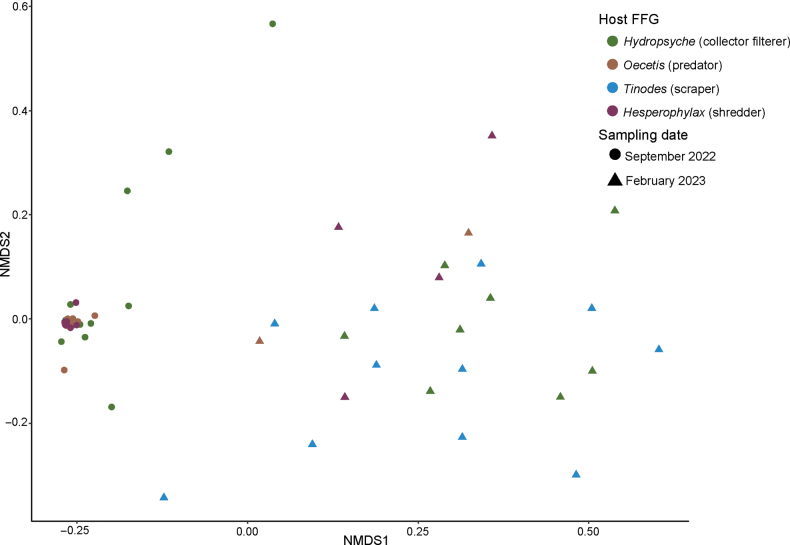
NMDS plot based on Bray–Curtis dissimilarity distances of gut microbiome composition between sampling dates. Point proximity indicates more similarity in community composition. NMDS stress = 0.120582759937782.

When comparing both sampling dates, the PERMANOVA test showed significant differences in gut microbiome composition among caddisfly genera (*P* < 0.05; Suppl. material 2: table S5). The pairwise comparisons showed significant microbiome variation for *Tinodes* sp. compared to that of *Hydropsyche* sp. (collector filterer), *Hesperophylax* sp. (shredder), and *Oecetis* sp. (predator), and *Hydropsyche* sp. to that of *Oecetis* sp. (Suppl. material 2: table S5). When comparing the microbiome composition separately for each sampling date, there were no differences in microbiota composition among the different phylogenetic groups (Figs 2C, D, 3, Suppl. material 2: table S5).

### ﻿Microbiome differences across sampling dates

The microbiome composition of the larvae differed significantly between the two sampling dates (Figs 2B, 3). The PERMANOVA test (*P* = 0.001) revealed a statistically significant variation in gut community composition between the two sampling dates, producing the highest pseudo-F score compared to the other pairwise comparisons (i.e., FFG/taxonomy of host; Suppl. material 2: table S5). For instance, the September samples had a greater proportion of Enterobacteriales (44.77%) than the February (9.94%) samples (Fig. 2B). Additionally, Thermotogales comprised 8.71% of the community’s relative abundance in the February samples. Other bacterial orders that comprised 6% of the gut microbiome were Actinomycetales for both dates, and Rhizobiales, Rhodospirillales and Acidobacteria-6 iii1-15 for the February samples.

## ﻿Discussion

In this study, we evaluated the gut microbiome composition of caddisfly larvae, encompassing four genera and functional feeding groups. The microbiome composition of the caddisfly samples was primarily composed of Proteobacteria and Actinobacteria. Enterobacteriales was the most abundant order of gut microbe, a finding mirrored in other insects, such as medflies (Behar et al. 2008; Aharon et al. 2013; Augustinos et al. 2015). While Enterobacteriales was a predominant microbe for the fall samples, they were not as abundant in the winter samples. Actinomycetales were also highly abundant across both sampling periods. Actinobacteria have been previously reported as a dominant gut microbiome in *Spodoptera frugiperda*, a lepidopteran species (Han et al. 2023).

We found that the microbiome was most strongly influenced by the collection date and, to a lesser extent, by the genus/functional feeding group of the host. The shift in microbiome composition observed between the two sampling dates may be attributed to a combination of biological and technical variability influencing the community composition. For instance, the similarities in gut bacteria composition of the different larvae at each sampling date could reflect strong environmental determinants of microbiota composition, which could homogenize their microbiome community across taxa at a given point in time (Ramírez and Gutiérrez-Fonseca 2014). Seasonal changes in the composition of food sources, such as periphyton and leaf litter, as well as microbial communities associated with these food sources and the aquatic environment, may be important drivers of gut microbiome change in caddisflies (Zeglin 2015; Hao et al. 2020; Cereghetti et al. 2025). In addition, although caddisflies possess various adaptations for acquiring food, they can demonstrate low selectivity for food resources, which may also impact microbiome variation across taxa (Wiggins and Currie 1996). There was no significant difference between dissected gut and whole-body samples, suggesting that the surface microbiota of the insects may be relatively sparse (Suppl. material 2: table S5).

While sampling date had the strongest influence on microbiome composition, the gut microbiomes of scrapers, which are specialized feeders, differed from those of larvae belonging to other functional feeding groups (Barbour 1999). In particular, Thermotogales were higher in relative abundance in the scraper that we sampled, *Tinodes* sp. In aquatic environments, Thermotogales have been reported in biofilm streamer communities, particularly in thermal aquatic environments (Meyer‐Dombard et al. 2011). As a sedentary feeding specialist, *Tinodes* sp. primarily consumes biofilm on streambed surfaces, which could possibly contribute to this higher proportion of Thermotogales in its microbiome (Stief and Becker 2005; Ings et al. 2017; Terefe et al. 2018; Chen et al. 2022). However, *Tinodes* sp., was only sampled during the winter, possibly contributing to most of the observed compositional differences when the combined data were compared. More replicates for each taxon are likely necessary to more comprehensively characterize the influence that feeding behavior has on microbiome communities. Making microbiome comparisons using species-level determinations may also be critical, as different species within the same genus can have differing feeding behaviors, which may influence their microbiome. For example, a study on the feeding behavior of three *Hydropsyche* species found in the same river indicated that although all exhibited omnivorous feeding habits and seasonal changes in food intake, one species demonstrated higher levels of carnivory than the others, which could potentially contribute to differences in microbiome composition among species (Fuller and Mackay 1980).

Previous studies have shown that diet is critical in shaping the microbiome across insects (Wang et al. 2011; Colman et al. 2012; Munoz-Benavent et al. 2021); however, the role that diet plays in shaping the microbiomes of aquatic insects is not well studied. Given their diversity in feeding modes, understanding whether the microbiota of caddisflies varies across feeding types is a crucial first step toward better understanding this potential relationship in aquatic insects (Merritt et al. 2017). While we have generated a first glimpse into the microbiomes of caddisflies in this experiment, future studies should consider sampling more widely and intensively across the order and include multiple caddisfly taxa within functional feeding groups to distinguish between the effect of host phylogeny and feeding type. A previous study on Lepidoptera (butterflies and moths) larvae, the sister order to Trichoptera, found a lack of a resident microbiome in their gut, indicating that the characterized microbiome was transient, likely influenced by the microbiome of their food and environment and played a reduced functional role (Hammer et al. 2017, 2019). Assessing whether this is also the case for caddisflies is crucial to understanding the function of the gut microbiome in this diverse aquatic insect order. Additionally, further insights could be gained using a finer taxonomic resolution (i.e., species) to unravel potential specific associations among species, feeding behaviors, and gut microbiome composition. Lastly, other biological and environmental factors, such as habitat type and life stage, that may also shape the microbiome in aquatic insects should be considered (Castillo et al. 2020; Nobles and Jackson 2020).

## ﻿Conclusion

In this study, we offered a preliminary characterization of the gut microbiome composition of caddisflies across functional feeding groups. While the greatest influence on microbiome composition was sampling date, the microbiome composition of scrapers in the genus *Tinodes* varied from other genera that we sampled. Future studies should consider including species-level classification and feeding information of larvae, more sampling replicates across time and additional taxa within different functional feeding groups, assessment of the degree of functionality and residency of the microbiome to better understand the impact and role of diet and feeding behaviors on the gut microbiome composition of aquatic larvae.

## ﻿Additional information

### Use of AI

No use of AI was reported.

### Author ORCIDs

Gabriela Jijón https://orcid.org/0000-0002-1893-3904

Danyon Gedris https://orcid.org/0009-0009-1635-1044

Paul B. Frandsen https://orcid.org/0000-0002-4801-7579

### Data availability

Sequencing reads are available under BioProject PRJNA1260018.

## Supplementary materials

Supplementary material 1 An image depicting a 1% agarose gel electrophoresis used to verify amplification success Data type docx https://binary.pensoft.net/file/1488935This dataset is made available under the Open Database License (http://opendatacommons.org/licenses/odbl/1.0/). The Open Database License (ODbL) is a license agreement intended to allow users to freely share, modify, and use this Dataset while maintaining this same freedom for others, provided that the original source and author(s) are credited.Author: Gabriela Jijón

Supplementary material 2 Supplementary information Data type xlsx https://binary.pensoft.net/file/1488936This dataset is made available under the Open Database License (http://opendatacommons.org/licenses/odbl/1.0/). The Open Database License (ODbL) is a license agreement intended to allow users to freely share, modify, and use this Dataset while maintaining this same freedom for others, provided that the original source and author(s) are credited.Author: Gabriela Jijón
