# Supplementary material for: ﻿Gut microbiome composition of Trichoptera larvae across functional feeding groups: a case study from the Provo River, Utah, USA
Source: Zookeys. 2025 Dec 10;1263:165–77. doi: 10.3897/zookeys.1263.147980 (PMC12712634; doi:10.3897/zookeys.1263.147980)
Supplement: Supplementary material 1 — An image depicting a 1% agarose gel electrophoresis used to verify amplification success [file zookeys-1263-165_article-147980__-s001.docx]

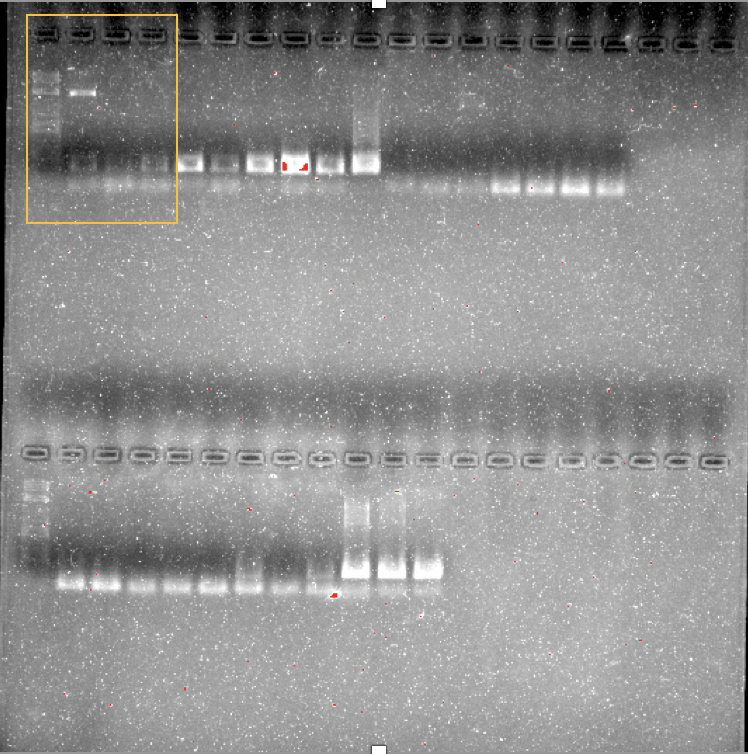


Figure S1. 1% agarose gel electrophoresis on a subset of samples (enclosed in the orange square) to assess the amplification success of the February 2023 samples.
